# Effectiveness of a Brief Point of Care Ultrasound Course at a National Nephrology Conference

**DOI:** 10.24908/pocus.v9i1.16858

**Published:** 2024-04-22

**Authors:** Abhilash Koratala, Nilam J Soni, Rupal Mehta, Nathaniel Reisinger

**Affiliations:** 1 Division of Nephrology, Medical College of Wisconsin Milwaukee, WI USA; 2 Department of Medicine, University of Texas Health San Antonio San Antonio, TX USA; 3 Division of Nephrology, Department of Medicine, Northwestern University Chicago, IL USA; 4 Renal Electrolyte and Hypertension Division, University of Pennsylvania Philadelphia, PA USA

**Keywords:** Ultrasound, Nephrology, Training, Workshop, Competency

## Abstract

The rising demand for point of care ultrasound (POCUS) instruction during nephrology fellowship has been limited due to a shortage of trained faculty and courses designed specifically for nephrologists. A hands-on POCUS pre-course was organized during the April 2023 National Kidney Foundation (NKF) Spring Clinical Meeting to address this challenge. The course consisted of pre-recorded lectures and a 4-hour hands-on workshop guided by multidisciplinary POCUS experts. The anonymous post-course survey received responses from 25 out of 39 participants, yielding a 64.1% response rate. On a scale of 0-10, confidence levels for acquiring kidney images rose from 2.6 + 2.3 (mean + SD) pre-workshop to 7.8 + 1.5 post-workshop (p<0.001). Similarly, a remarkable improvement in confidence for acquiring lung and cardiac images was seen as scores increased from 1.8 + 2.4 to 7.7 + 1.5 (p<0.001) and from 1.5 + 2.2 to 7.2 + 1.3 (p<0.001), respectively. Additionally, respondents reported a substantial improvement in their confidence to interpret kidney, lung, and cardiac POCUS images, with scores increasing from 4.5 + 2.2 to 7.7 + 1.1 (p<0.001), 2.3 + 2.4 to 7.6 + 1.5 (p<0.001), and 2 + 2 to 7.3 + 1.5 (p<0.001), respectively. Barriers to implementing POCUS use at institutions included a perceived lack of trained faculty, limited protected time for faculty, and insufficient support from division leadership. The NKF POCUS pre-course successfully improved participants’ confidence in acquiring and interpreting basic POCUS images.

## Introduction

Point of care ultrasound (POCUS) refers to clinician-performed, focused ultrasound exams to answer specific clinical questions at the bedside that guide management 1. Over the past 25 years, POCUS has emerged as a powerful tool to improve clinicians’ diagnostic accuracy when evaluating patients in most specialties. In nephrology, POCUS is no longer confined to kidney ultrasound or procedural guidance but includes a broad range of applications, including assessment of volume status, cardiopulmonary disease, and venous thrombosis 2, 3. Most medical schools (78%) have integrated structured POCUS training into their preclinical and clinical curricula 4, and 61% of internal medicine residency programs had a POCUS curriculum in 2020 5. An increasing number of nephrology fellows are starting fellowship with POCUS skills that their supervising attending nephrologists may lack. As such, it is now imperative that practicing nephrologists and graduating fellows have a working knowledge of POCUS imaging. To help bridge this training gap, a hands-on POCUS pre-course was conducted at the National Kidney Foundation (NKF) Spring Clinical Meeting in April 2023. The goal of the course was to introduce foundational knowledge and hands-on skills for nephrologists in-practice to perform basic POCUS exams pertinent to nephrology. Here we describe the course and summarize findings of a post-course survey that assessed course effectiveness.

## Materials and Methods

**Figure 1:**
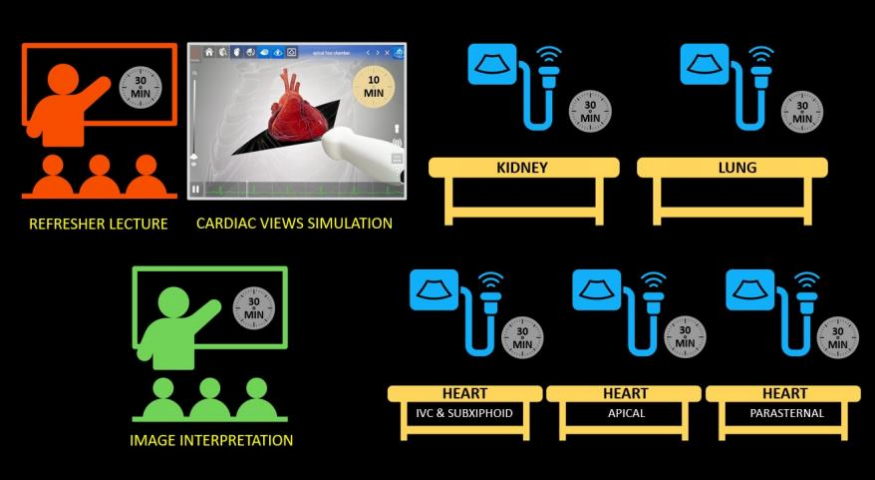
Nephrology Point of Care Ultrasound Workshop Curriculum. Two identical half-day courses were conducted. IVC, inferior vena cava.

A set of 5 pre-recorded lectures lasting 15-20 minutes each was provided to learners as required pre-course work. This approach maximized time for hands-on scanning and expert-guided image review. Two identical 4-hour POCUS courses were offered on the same day to allow small group scanning sessions with 4 learners and 1 faculty per station; maximizing learner engagement and offering flexibility in attending the course (Figure 1). Six POCUS experts (2 nephrologists, 2 hospitalists, 1 nephrologist-intensivist, and 1 intensivist) served as faculty. The course began with an introductory lecture reinforcing the basics of image orientation and acquisition. Next, a short cardiac anatomy simulation using Heartworks® augmented reality simulator was used to demonstrate 3-dimentsional cardiac anatomy and basic cardiac POCUS views. For hands-on practice, learners rotated through 5 scanning stations equipped with a portable ultrasound machine and live model. Each station was dedicated to teaching a specific POCUS application – one station for kidneys and bladder, one station for lungs and pleura, and three stations for cardiac views (parasternal, apical, and subcostal views). More time was allocated for cardiac POCUS since it is a crucial component of volume status assessment and acquiring proficiency can be challenging due to the complexity of cardiac anatomy. Learners practiced acquiring standard views on healthy models under the guidance of expert faculty and rotated through each station every 30 minutes. The workshop concluded with a 35-minute image review session showing common POCUS abnormalities relevant to nephrology. Following the conclusion of the meeting, an anonymous and voluntary post-course survey was distributed via email to collect feedback and evaluate the effectiveness of the course. No demographic data were collected, except for the participants' professional roles. Participants' confidence to perform and interpret POCUS applications taught in the course was evaluated on a 10-point Likert scale. Likewise, participants were requested to rate the perceived barriers to implement POCUS use at their institutions, utilizing a 10-point scale (0=no hindrance; 10=complete roadblock). Analyses were performed using GraphPad Prism version 10.0.1 for Windows, provided by GraphPad Software, San Diego, CA, USA (www.graphpad.com). The confidence levels were reported using the mean and standard deviation, assuming a normal distribution of the data.

## Results

**Figure 2:**
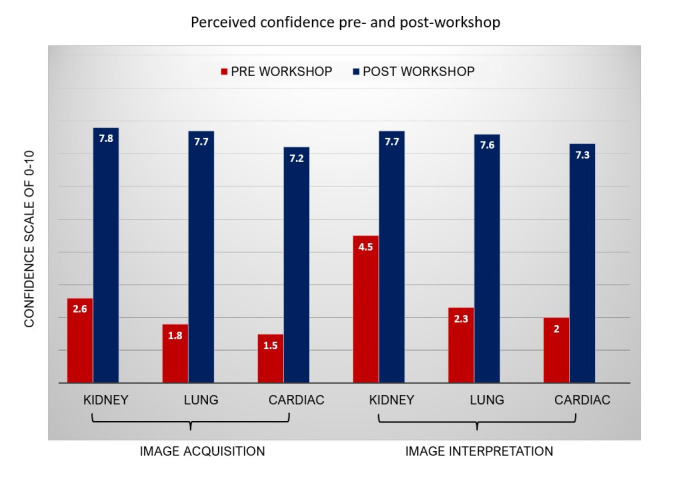
Pre- and Post-course Confidence of Learners in POCUS Image Acquisition and Interpretation.

A total of 39 learners attended the course (16 in the morning; 23 in the afternoon) and 25 unique learners completed the post-course survey for a response rate of 64%. Among learners completing the post-course survey, 63% were practicing nephrologists, 23% were nephrology fellows, and 14% were advanced practice providers and internal medicine residents. Learners rated their level of confidence in acquiring and interpreting POCUS images on a 10-point Likert scale. Comparing pre- and post-course ratings, learners' confidence in acquiring renal images increased significantly from a mean of 2.6 (± 2.3) pre-course to 7.8 (± 1.5) post-course (p<0.001). Similarly, learner confidence in acquiring lung and cardiac images increased from 1.8 (±2.4) to 7.7 (± 1.5) (p<0.001) and from 1.5 (±2.2) to 7.2 (± 1.3) (p< 0.001), respectively. With respect to confidence in image interpretation, learners reported a substantial improvement in interpreting kidney, lung, and cardiac POCUS images from 4.5 (±2.2) to 7.7 (±1.1) (p<0.001), 2.3 (±2.4) to 7.6 (± 1.5) (p<0.001), and 2 (±2) to 7.3 (±1.5) (p<0.001), respectively (Figure 2). Regarding course duration, 81% of learners felt the duration was appropriate, while 15% would have preferred a longer course. Interestingly, all learners responded that the cardiac anatomy simulation improved their understanding of cardiac POCUS anatomy. The top 3 perceived barriers to implementing POCUS use at the learners’ institutions (based on a subjective rating scale of 1-10), were lack of trained faculty (mean score of 7.5), lack of protected time for faculty (7.2), and lack of support from their division leadership (6.8).

## Discussion

The spring 2023 NKF POCUS pre-course was successful at improving the confidence of nephrology attending physicians and fellows in acquiring and interpreting common POCUS views relevant to nephrology. Although the substantial boost in learner confidence is encouraging, it may be influenced by their excitement to learn new skills. This increased confidence does not automatically translate into competency. Achieving competence requires structured, longitudinal practice, and honing skills acquired during workshops like this can serve as a starting point. In fact, studies have consistently demonstrated that confident learners may not necessarily be competent, especially in the absence of continued supervised practice 6, 7. As the shortage of qualified nephrology faculty poses a significant obstacle to widespread POCUS adoption 8, nephrology professional societies should take a leading role to deliver sustained and methodical POCUS training to interested nephrologists. Notably, all learners said they would (89%) or may (11%) pursue a longitudinal POCUS training program if NKF offered such a program. Furthermore, publication of specialty-specific guidelines by respected organizations delineating the scope of practice, training requirements, and competency standards for nephrologists would garner institutional support and help standardize practice. Without such initiatives, an increasing disparity between the desire to incorporate POCUS into practice and availability of POCUS-trained nephrology attending physicians may lead to POCUS use that lacks quality and standards.

## Conflict-of-Interest statement

The authors declare no potential conflicts of interest.

## Funding statement

AK reports research funding from KidneyCure and the American Society of Nephrology’s William and Sandra Bennett Clinical Scholars Grant. NS reports receiving grant funding from the Department of Veterans Affairs Quality Enhancement Research Initiative (QUERI) Partnered Evaluation Initiative (I50 HX002263-01A1) and National Center for Patient Safety. The contents of this publication do not represent the views of the US Department of Veterans Affairs or the US Government. RM is supported by grant K23HL150236. RM owns stock in Abbvie and has received consultation fees/honoraria from AstraZeneca and is on the Spealer’s Bureau for AstraZeneca.

## Ethics Approval

 This study falls outside the scope of the institutional review board. The results have been shared with the meeting/course organizers and approved for publication.

## Data Availability Statement

 Available data is furnished in the manuscript. Further enquiries can be directed to the corresponding author.

## Author Contributions

All authors contributed to drafting the manuscript and organization of the course. AK served as course director. All authors have seen and approved the final version of the manuscript.

## Acknowledgements

We thank Jordan Cannon, Sr. Director, Professional Programs & Member Engagement, NKF for the help and support in organizing this workshop. We are grateful to all the course faculty for sharing their expertise.
